# Isotopic H/D Exchange in Hydrogen Bonds Between the Nitrogenous Bases of the CAG Repeat Tract Makes It Possible to Stabilize Its Expansion in the *ATXN2* Gene

**DOI:** 10.3390/biomedicines13112708

**Published:** 2025-11-04

**Authors:** Anna Dorohova, Luis Velázquez-Pérez, Mikhail Drobotenko, Oksana Lyasota, Jose Luis Hernandez-Caceres, Roberto Rodriguez-Labrada, Alexandr Svidlov, Olga Leontyeva, Yury Nechipurenko, Stepan Dzhimak

**Affiliations:** 1 Scientific Department, Kuban State University, 350040 Krasnodar, Russia; 2 Laboratory of Problems of Stable Isotope Spreading in Living Systems, Southern Scientific Center of the Russian Academy of Sciences, 344006 Rostov-on-Don, Russia; 3 President’s Office, Cuban Academy of Sciences, La Habana 10100, Cuba; 4 Neurodevelopment Branch, Cuban Center for Neurosciences, La Habana 11600, Cuba; 5 Joint Institute of Nuclear Research, 141980 Dubna, Russia; 6 Engelhardt Institute of Molecular Biology, Russian Academy of Sciences, 119991 Moscow, Russia

**Keywords:** *ATXN2* gene, CAG repeat tract, H/D exchange, SCA2, parkinsonism, mathematical modeling, open states, torque

## Abstract

**Background**: The isotopic composition of the body’s internal environment can affect its functional state. Such effects are realized, among other things, by inserting deuterium atoms into hydrogen bonds between pairs of nitrogenous bases of DNA molecules and modifying their mechanical properties. **Methods**: This study uses a coarse-grained mathematical model of DNA. **Results**: It has been established that in a certain range of the magnitude of the torque, with the presence of a deuterium atom within it, stabilization of the CAG repeat tract is observed. In addition, it was found that, regardless of which base pair the deuterium atom falls into in the CAG repeat tract, its stability increases and the probability of hairpin formation decreases, which may interfere with the reading of genetic information from the site encoding glutamine. **Conclusions**: Single H/D substitutions in the CAG repeat tract of the *ATXN2* gene increase its stability by reducing the formation of open states, regardless of the position of deuterium.

## 1. Introduction

The *ATXN2* gene contains a CAG repeat tract, which encodes the polyglutamine tract (polyQ) in the ATXN2 protein. In healthy people, the CAG repeat tract usually contains 22–23 CAG [1,2,3,4,5]. CAG expansion in *ATXN2* causes a spectrum of diseases from SCA2 to ALS, which makes the gene an important target for neurodegeneration studies [6,7,8,9]. Type 2 ataxia manifests itself in the following pathologies in late-stage dementia: impaired coordination, slow eye movements, and tremor [10,11,12,13].

In the longer CAG repeat tract, the disease begins earlier and the symptoms are more severe [14,15,16,17]. The following clinical facts were found: correlation between the length of the CAG repeat tract and the age of onset of the disease [18,19].

CAG repeats tend to form unstable secondary structures (hairpins). CAG repeats form loops during replication/repair, which promote DNA polymerase errors and their expansion [20,21,22,23,24,25]. In addition, a correlation was established by computer modeling between the value of the probability of additional zones of open states in the CAG repeat tract and clinical data on the age of onset of the disease [26].

In cases of parkinsonism, it was found that in the CAG repeat tract, CAG repeats are interrupted by one or more CAA inserts [27,28,29,30,31].

CAA interruption reduces the tendency for further repeat elongation in the next generations. They can disrupt the formation of pathogenic secondary DNA structures (for example, hairpins), slowing down the expansion of trinucleotide repeats [32,33,34,35,36,37,38,39]. Patients with the same length of CAG repeat tract but a different number of CAA interruptions may have differences in the age of onset of the disease [28,40]. The CAA interruption is the substitution of the G nucleotide for an A in one of the CAG trinucleotides. This modification leads to a change in the mechanical properties of the nucleotide sequence, as well as a significant change in the energy of complementary hydrogen bonds. At first glance, such a replacement should reduce the stability of the CAG repeat tract, but the calculated data demonstrate a paradoxical effect—some positions of CAA interruptions stabilize the CAG repeat tract. We have found that interruptions located near the center of the CAG repeat tract significantly reduce its stability, and those located near its boundaries can both reduce and increase the stability of the CAG repeat tract. At the same time, there is a certain asymmetry: CAA interruptions located near the left border (near the promotor) of the CAG repeat tract have a more stabilizing effect than CAA interruptions located near the right border [41,42,43].

This phenomenon highlights the key role of DNA dynamics, in which even a single nucleotide substitution can significantly affect the overall stability of the trinucleotide repeat zone [44].

From the point of view of pharmacological perspectives, a logical question arises: is it possible to increase the stability of the CAG repeat tract by strengthening H bonds? The most physiological way to do this is by isotopic substitution of protium/deuterium (H/D) in base pairs. It is known that the deuterium bond is about 5% stronger than the hydrogen bond [45]; therefore, the H/D substitution strengthens the hydrogen bonds in the pairs of nitrogenous bases.

The aim of this work is to study the effect of single H/D substitutions on the stability of the CAG repeat tract, depending on the deuterium localization.

The study was carried out using computer modeling based on a mechanical DNA model, which allows us to accurately describe the processes occurring in DNA [46,47,48,49,50,51].

## 2. Materials and Methods

### 2.1. Mathematical Model

A mechanical model for describing the rotational movements of DNA bases was built by Englander and co-authors in 1980 [52]. The model is based on an analogy between a double-stranded DNA molecule and a mechanical system consisting of two chains of connected pendulums that rotate in a plane that is orthogonal to the axis of the chains. The angular mathematical model of DNA was most fully developed in the works of Yakushevich [53]. This model considers the heterogeneity of the DNA molecule. Using a wide range of external influences, it allows us to describe zones of open states (OSs), defined as mobile areas (ranging in size from one to several base pairs), inside which hydrogen bonds are broken [54,55,56,57,58].

For our research, we use an upgraded model, of which the mathematical formulation is a system of nonlinear ordinary differential equations with respect to the angular deflections of pendulums [59]:

(1) $$I_{1}^{i}\frac{d^{2}\varphi_{1}^{i}(t)}{dt^{2}}=K_{1}^{i}\left[\varphi_{1}^{i-1}(t)-2\varphi_{1}^{i}(t)+\varphi_{1}^{i+1}(t)\right]\;-\delta^{i}\left(k_{12}^{i}R_{1}^{i}(R_{1}^{i}+R_{2}^{i})sin\varphi_{1}^{i}+k_{12}^{i}R_{1}^{i}R_{2}^{i}sin(\varphi_{1}^{i}-\varphi_{2}^{i})\right)+F_{1}^{i}\left(t\right),\;i=\bar{2,n-1},$$

(2) $$I_{1}^{1}\frac{d^{2}\varphi_{1}^{1}\left(t\right)}{dt^{2}}=K_{1}^{1}\left[\varphi_{1}^{2}\left(t\right)-\varphi_{1}^{1}\left(t\right)\right]\;-\delta^{i}\left(k_{12}^{1}R_{1}^{1}(R_{1}^{1}+R_{2}^{1})sin\varphi_{1}^{1}+k_{12}^{1}R_{1}^{1}R_{2}^{1}sin(\varphi_{1}^{1}-\varphi_{2}^{1})\right)+F_{1}^{1}(t),$$

(3) $$I_{1}^{n}\frac{d^{2}\varphi_{1}^{n}(t)}{dt^{2}}=K_{1}^{n}\left[\varphi_{1}^{n-1}(t)-\varphi_{1}^{n}(t)\right]\;-\delta^{i}\left(k_{12}^{n}R_{1}^{n}\left(R_{1}^{n}+R_{2}^{n}\right)sin\varphi_{1}^{n}+k_{12}^{n}R_{1}^{n}R_{2}^{n}\sin\left(\varphi_{1}^{n}-\varphi_{2}^{n}\right)\right)+F_{1}^{n}\left(t\right),$$

(4) $$I_{2}^{i}\frac{d^{2}\varphi_{2}^{i}(t)}{dt^{2}}=K_{2}^{i}\left[\varphi_{2}^{i-1}(t)-2\varphi_{2}^{i}(t)+\varphi_{2}^{i+1}(t)\right]\;+\delta^{i}\left(k_{12}^{i}R_{2}^{i}\left(R_{1}^{i}+R_{2}^{i}\right)sin\varphi_{2}^{i}-k_{12}^{i}R_{1}^{i}R_{2}^{i}\sin\left(\varphi_{2}^{i}-\varphi_{1}^{i}\right)\right)++F_{2}^{i}\left(t\right),\;i=\bar{2,n-1},$$

(5) $$I_{2}^{1}\frac{d^{2}\varphi_{2}^{1}\left(t\right)}{dt^{2}}=K_{2}^{1}\left[\varphi_{2}^{2}\left(t\right)-\varphi_{2}^{1}\left(t\right)\right]\;{+\delta }^{i}\left(k_{12}^{1}R_{2}^{1}(R_{1}^{1}+R_{2}^{1})sin\varphi_{2}^{1}1-k_{12}^{1}R_{1}^{1}R_{2}^{1}sin(\varphi_{2}^{1}-\varphi_{1}^{1})\right)+F_{2}^{1}(t),$$

(6) $$I_{2}^{n}\frac{d^{2}\varphi_{2}^{n}(t)}{dt^{2}}=K_{2}^{n}\left[\varphi_{2}^{n-1}(t)-\varphi_{2}^{n}(t)\right]\;+\delta^{i}\left(k_{12}^{n}R_{2}^{n}(R_{1}^{n}+R_{2}^{n})sin\varphi_{2}^{n}-k_{12}^{n}R_{1}^{n}R_{2}^{n}sin(\varphi_{2}^{n}-\varphi_{1}^{n})\right)+F_{2}^{n}(t).$$

Here,

$I_{j}^{i}$—Moment of inertia of the *i*-pendulum of the *j*-chain;

$R_{j}^{i}$—Distance from the center of mass of the *i*-pendulum of the j-chain to the thread;

$\varphi_{j}^{i}(t)$—Angular deviation of the *i*-pendulum of the *j*-chain, counted counterclockwise, at time *t*;

$k_{12}^{i}$—Constant characterizing the elastic properties of the connection of the *i*-pair of pendulums;

$K_{j}^{i}$—Constant characterizing the torque of the *i*-section of the *j*-thread;

$F_{j}^{i}\left(t\right)\;$= $-\beta_{j}^{i}\frac{d\varphi_{j}^{i}}{dt}(t)$ + $M^{i}(t)$—External influence on the *i*-pendulum of the j-chain at time *t*;

n—The number of pairs of pendulums in the system.

The first term to the right of the equal sign in differential Equations (1)–(6) describes the force action on the *i*-th pendulum from the elastic sugar-phosphate chain, the second term is the influence from the paired pendulum, the value of $-\beta_{j}^{i}\frac{d\varphi_{j}^{i}}{dt}(t)$ allows us to describe the effects of dissipation—which are caused by the interaction between the DNA molecule and the surrounding liquid—and $M^{i}(t)$ is an external torque.

Equations (1)–(6) allow us to model the hydrogen bond in the *i*-th base pair ($\delta^{i}=1$, $k_{12}^{i}=k_{12}^{H,i}$), deuterium bond ($\delta^{i}=1$, $k_{12}^{i}=k_{12}^{D,i}$) and its breaking ($\delta^{i}=0$) [60]. A hydrogen bond break in the *i*-th base pair occurs if the potential binding energy in this pair exceeds the critical value, which is equal to *E_AT_* for the AT pair and *E_GC_* for the GC pair; the bond is restored if its potential energy becomes less than the critical value.

We add the initial conditions to Equations (1)–(6):

(7) $$\varphi_{1}^{i}\left(0\right)=\varphi_{1,0}^{i},\frac{d\varphi_{1}^{i}}{dt}\left(0\right)=\varphi_{1,1}^{i},$$

(8) $$\varphi_{2}^{i}\left(0\right)=\varphi_{2,0}^{i},\frac{d\varphi_{2}^{i}}{dt}\left(0\right)=\varphi_{2,1}^{i},i=\bar{1,n},$$

The values of the parameters in Equations (1)–(6) are taken from the works [53,54,61] and are shown in Table 1.

The values of the energy of hydrogen bond breaking in pairs AT and GC are taken from the following [62]: *E_AT_* = 5.1020 pN · nm, *E_GC_* = 12.7064 pN · nm.

Using conditions (7)–(8), an undisturbed state was set, i.e., $\varphi_{1,0}^{i}=\varphi_{1,1}^{i}=\varphi_{2,1}^{i}=0,\varphi_{2,0}^{i}=\pi ,i=\bar{1,n}.$

The torsion effect of *M^i^(t)* was chosen to be constant in time and spatially localized over the interval [*i*_1_, *i*_2_], i.e., $M^{i}\left(t\right)=M_{0}^{i}$, $i=\bar{1,n}$; moreover, *M^i^*_0_ *= M*_0_ at 1 ≤ *i*_1_ ≤ *i* ≤ *i*_2_ ≤ *n* and *M^i^*_0_ = 0 for other *i* values.

The system of differential equations with initial conditions (1)–(8) was solved numerically by the Runge–Kutta method, using the original program [63]. The comparison of the computer’s simulation results with the experimental data on single DNA unwinding [64,65] showed the adequacy of the proposed model.

### 2.2. Description of the Numerical Experiment

The *ATXN2* gene contains more than 130,000 base pairs. Approximately 2000 base pairs in the first exon are essential for studying the processes in the CAG repeat tract. Therefore, a region of the *ATXN2* gene containing a CAG repeat tract and including nucleotides numbered from 4601 to 6600 at 23 CAG repeats was selected for calculations [26]. With this choice, the gene region contains the first exon, and at the same time, the disturbance zone caused by the applied torque is located within the selected region for an estimated period of time. This makes it possible to select the condition of absence of disturbances as boundary conditions for the calculated DNA segment. If the size of the CAG repeat tract increased during the calculations, then the right border of the calculated section increased accordingly.

Previous studies have shown that at values of the external torsion moment *M*_0_ ≥ 8.28 pN · nm, significant OS zones are formed in the promoter region of the isolated DNA area. In addition, at these values of *M*_0_, OS zones of various sizes can additionally form in the CAG repeat tract. We will characterize the stability of the CAG repeat tract by calculating the probability of the formation of additional large OS zones in it under torque *M*_0_ ≥ 8.28 pN · nm. If the probability is high, we will talk about the low stability of the CAG repeat tract and the reverse.

Calculations have shown that in the extended CAG repeat tract, significant OS zones are formed in the promoter region at *M*_0_ ≥ 8.28 pN · nm. An increase in the value of the torque *M*_0_ can lead to the formation of additional open state zones in the CAG repeat tract.

It was previously shown that with the increasing length of the CAG repeat tract in the *ATXN2* gene, its stability decreases; this increases the probability of additional large OS zones’ occurrence in it [26]. A correlation was established between this probability and statistical data on the average age of SCA2 disease onset [66,67]. It should be noted that additional OS zones in the CAG repeat tract increase the probability of secondary structure formation and prevent the reading of information from DNA [26,68,69,70].

Study of the hydrogen bonds’ potential energy dynamics in base pairs in the *ATXN2* gene showed that the oscillatory movements of DNA under the torque lead to a redistribution of the hydrogen bonds’ potential energy and its concentration in places located in the CAG repeat tract [71]. These processes can cause the formation of additional OS zones in the CAG repeat tract and affect its stability.

The CAG repeat tract is highly sensitive to changes in its parameters. Studies on the effect of CAA interruptions on the stability of the CAG repeat tract have shown that single interruptions (which led to small changes in the mechanical properties and magnitude of hydrogen bonds in one pair of CAG repeat tract nucleotides) can significantly affect the stability of the CAG repeat tract. Moreover, an increase or decrease in its stability depends on the location of the interruption [37,42].

The authors believe that the main influence of CAA interruptions on the stability of the CAG repeat tract is a change in the energy of hydrogen bonds in the substituted base pair. In this regard, the following question arises: is it possible to directly affect the stability of the CAG repeat tract by slightly changing the energy of hydrogen bonds in a base pair without changing its mechanical properties?

Such changes can be modeled with D/H substitution in the nucleotides of the CAG repeat tract. In this case, we assume that a single D/H substitution leads to an increase in the energy of one of the hydrogen bonds in the base pair by 5% [45]; we will ignore the change in the moment of inertia in the base during such a replacement.

### 2.3. Calculating the Probabilities of Open States Formation

The parameters for calculating the probability of additional large OS zones in the CAG repeat tract will be the same as for a pure CAG tract [26] and for single CAA interruptions [42]. The value of the torque *M*_0_ varied from 8.28 pN · nm (the beginning of OS formation in the promoter zone) to 8.62 pN · nm, the segment of localization of the torsion effect [*i*_1_, *i*_2_] was set so that the left boundary of *i*_1_ corresponded with the beginning of the promoter zone (*i*_1_ = 633rd base pair from the beginning of the isolated DNA region), and the right boundary of *i*_2_ varied from the 1170th to 1320th base pair from the beginning of the selected segment.

To calculate the probability of additional large OSs zone formation, the calculation results were summarized in tables that characterize the size of additional OS zones: 0 in the table means that with this set of parameters, there is no additional OSs zone in the CAG repeat tract or it is very small, and 1 means that the additional OSs zone is large.

## 3. Results and Discussion

### 3.1. Examples of Additional OS Zones Formation in the CAG Repeat Tract

**Figure 1:**
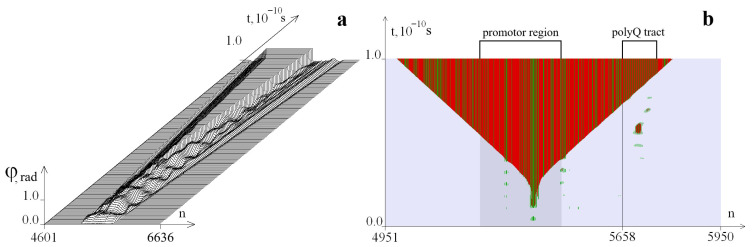
Angular deviations of the first chain (**a**) and the OSs zone (**b**) that occur during torsion action on the segment of the *ATXN2* gene at *M*_0_ = 8.28 pN · nm. The numbers of the pairs of nitrogenous bases are set horizontally, and the time is set vertically. For clarity, the amplitude of the angular deviations is limited to 0.5 rad. The OSs in pairs AT are indicated in green, and the GC pairs in red. The promoter region is highlighted with a darker background. The beginning of the CAG repeat tract is the 5658th base pair.

**Figure 2:**
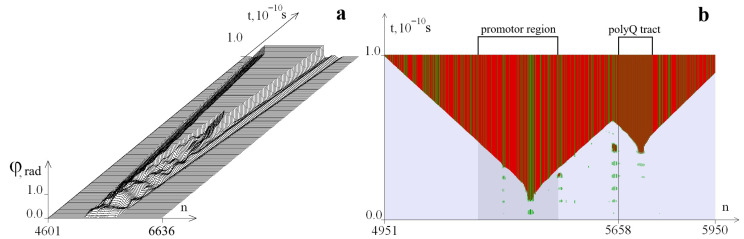
Angular deviations of the first chain (**a**) and the OSs zone (**b**) that occur during torsion action on the *ATXN2* gene segment at *M*_0_ = 8.50 pN · nm. The numbers of the pairs of nitrogenous bases are set horizontally, and the time is set vertically. For clarity, the amplitude of the angular deviations is limited to 0.5 rad. The OSs in pairs AT are indicated in green, and the GC pairs are in red. The promoter region is highlighted with a darker background. The beginning of the CAG repeat tract is the 5658th base pair.

Here are examples of the formation of OSs zones in the selected segment of the *ATXN2* gene with the number k = 35 of trinucleotide repeats in the CAG repeat tract. Figure 1 and Figure 2 show graphs of the angular deviations of the first chain of nitrogenous bases (for clarity, the amplitude is limited to 0.5 rad) and OSs at different values of the torque M_0_. In this case, for the left boundary of the torsion effect (counted from the beginning of the selected gene region), *i*_1_ = 633 corresponds to the beginning of the promoter region, and the right boundary was *i*_2_ = 1205. The time interval [0, 10^−10^ s] was selected for the calculation. The OSs in pairs AT are highlighted in green in all figures, and the GC pairs are in red. The promoter region is highlighted in dark gray, and the beginning of the CAG repeat tract (5658th base pair) is indicated by a line. The numbers of the pairs of nitrogenous bases are plotted along the horizontal axis, and time is plotted along the vertical axis.

Figure 1 is constructed for *M*_0_ = 8.28 pN · nm. In Figure 1a, it can be seen that the disturbance does not reach the boundaries of the selected segment; Figure 1b shows that a large OSs zone has appeared in the promoter region, and no large OS zones have formed in the CAG repeat tract. Figure 2 is constructed for *M*_0_ = 8.50 pN · nm. Figure 2b shows that in addition to the large OSs zone in the promoter region, an additional large OSs zone was formed in the CAG repeat tract.

Figure 1a,b show that a large OSs zone has appeared in the promoter region. The large OSs zone in the CAG repeat tract did not form. In the tables, it is assigned the symbol 0.

Figure 2a,b show that large OSs zones were formed in the promoter region and in the CAG repeat tract. We call the OSs zone in the CAG repeat tract an additional large OSs zone, and the symbol 1 is assigned to it in the tables.

### 3.2. The Probability of Additional Large OSs Zone Formation Depending on the Localization of the H/D Substitution in the CAG Repeat Tract

**Figure 3:**
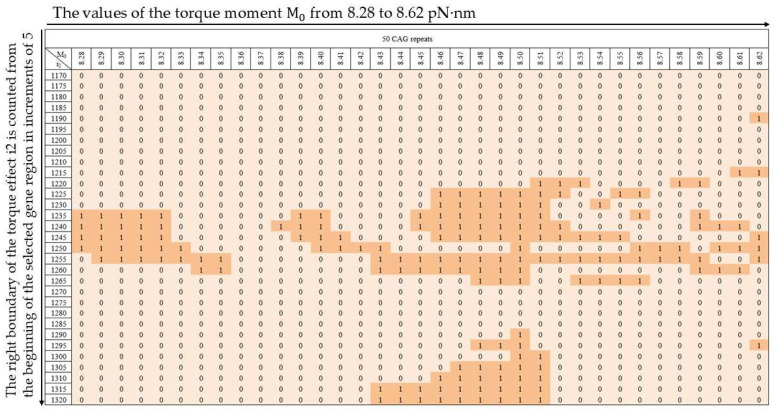
Table of additional OSs zones for the CAG repeat tract with the number of trinucleotide repeats being k = 50, in which the hydrogen bond in the base pair corresponding to the cytosine of the fifth trinucleotide is replaced by deuterium. The values of the right boundary of the torque i2 are set vertically in the table and the values of the M0 are set horizontally. In the table, 0 means that with this set of parameters, the additional OSs zone in the CAG repeat tract is absent or very small, and 1 means that the additional OSs zone is large; these values in the table are additionally highlighted in color.

The probabilities of additional large OSs zone formation at deuterium localization in base pairs corresponding to each of the nucleotides of the m-th trinucleotide of the CAG (m = 5, 10, 15, 20, 25, 30, 35, 40, 45, 50) in the CAG repeat tract were calculated. For this purpose, the calculated results were summarized in tables of additional OSs zones. For example, Figure 3 summarizes additional OSs zones for the CAG repeat tract with the number of trinucleotide repeats being k = 50, in which the hydrogen bond in the base pair corresponding to the cytosine of the fifth trinucleotide is replaced by a deuterium bond. The rest of the tables are given in the Supplementary Materials Figure S1.

**Figure 4:**
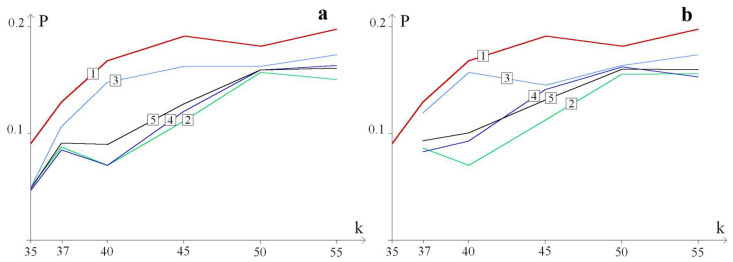
Graphs of the probability P of the additional large OSs zone formation, depending on the trinucleotides number (k) in the CAG repeat tract, with single H/D substitutions in the CAG repeat tract. Graph 1 corresponds to a CAG repeat tract that does not contain deuterium bonds. Graphs 2, 3, and 4 correspond to the CAG repeat tract in which the hydrogen bond in the base pair corresponding to cytosine, adenine, and guanine of the m-th trinucleotide is replaced by a deuterium bond. Finally, 5 is a graph of the probability of the additional OSs zone formation with a single H/D substitution in the m-th trinucleotide (average value). (**a**) Shows graphs for m = 5, and (**b**) shows graphs for m = 35.

Figure 4 shows graphs of the probability, P, of the additional large OSs zone formation, depending on the number of k trinucleotides in the CAG repeat tract. The red graph marked 1 does not contain deuterium bonds. The remaining graphs correspond to the CAG repeat tract, in which the hydrogen bond in the base pairs is replaced by deuterium: Figure 4a in the base pairs of the 5th trinucleotide, and Figure 4b in the base pairs of the 35th trinucleotide.

The average probabilities of the formation of additional OSs zones during localization of the H/D substitution in the 5th and 35th trinucleotides were also calculated. Graphs of these probabilities are also shown in Figure 4. Graphs of the probability of additional OSs zone formation during localization of the H/D substitution in the remaining trinucleotides are given in the Supplementary Materials Figure S1.

Figure 4 shows that H/D substitutions stabilize the CAG repeat tract. At the same time, H/D substitutions in GC pairs stabilize the CAG repeat tract more strongly than in AT pairs. This can be explained by the fact that in most cases, openings in GC pairs are critical for the OSs zone formation. Therefore, the strengthening of hydrogen bonds in these pairs more strongly stabilizes the CAG repeat tract.

The calculations performed showed that the probability of the additional large OSs zones’ formation with single H/D substitutions in the CAG repeat tract depends very little on the number of the trinucleotides. The graphs of such probabilities are in a fairly narrow band.

**Figure 5:**
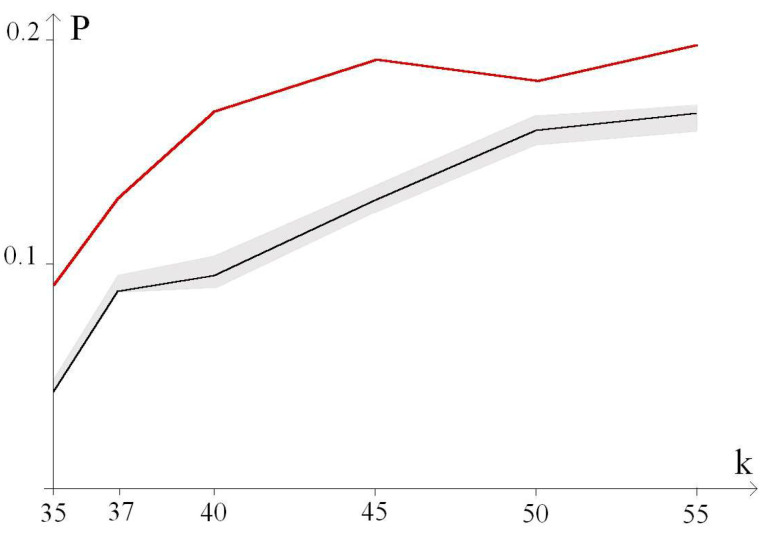
Graphs of the additional large OSs zone formation probability, P, depending on the number of k trinucleotides in the CAG repeat tract. The gray color shows the band containing the values of the additional large OS zone formation probabilities, P, with single H/D substitutions in the m-th trinucleotide (m = 5, 10, 15, 20, 25, 30, 35, 40, 45, 50) of the CAG repeat tract. The probability graph at m = 25 is highlighted in black. The graph of the additional large OSs zone formation probability for the CAG repeat tract that does not contain deuterium bonds is shown in red. The number of k trinucleotides in the CAG repeat tract is horizontally deposited.

Figure 5 shows a band containing graphs of the additional large OSs zone formation probability, P, with single H/D substitutions in the m-th trinucleotide (m = 5, 10, 15, 20, 25, 30, 35, 40, 45, 50) of the CAG repeat tract. The probability graph at m = 25 is highlighted in black. For comparison, a graph of the probability of the additional large OS zone formation for the CAG repeat tract that does not contain deuterium bonds is presented.

It can be seen from the above figures that a single H/D substitution in base pairs, which leads to a 5% strengthening of one of the hydrogen bonds in these nitrogenous bases, leads to the stabilization of the CAG repeat tract. At the same time, the degree of stabilization does not depend much on the localization of the H/D substitution. Thus, the effect of the H/D substitution on the probability of additional large OS zone occurrence in the CAG repeat tract is determined only by the probability of deuterium entering the CAG repeat tract. Therefore, H/D substitution may be interesting as a mechanism for stabilizing the CAG repeat tract.

The proposed approach may be further developed when conducting experiments with low deuterated water in mouse models of spinocerebellar ataxia [72].

Coarse-grained DNA modeling is an approach that sacrifices atomic precision for computational efficiency. Because DNA is a very long molecule, its detailed modeling requires enormous resources. Coarse-grained models simplify the structure by reducing the number of details, which speeds up calculations by tens of times. This enables the study to use slow processes that are inaccessible for precise methods, such as DNA folding, chromatin dynamics, and protein interactions. The level of detail can be flexibly adjusted to suit a specific task—from studying short fragments to modeling entire chromosomes—making the method a universal tool in molecular biology and nanobiotechnology.

For studying open states in DNA, coarse-grained simulations are ideal because they deal with large molecular systems and long time periods that are not possible with full-atom simulations [73,74,75]. The formation and dynamics of OS zones in a double-stranded DNA molecule are largely determined by its mechanical properties, which are well reproduced in coarse-grained models [48,76,77].

The authors used the angular model of DNA, since its parameters are consistent with known experimental results: for example, the magnitude of the torsional effect corresponds to that measured in the work [64,65]. The coefficients of the equations were measured experimentally [53]. The energies of hydrogen bond dissociation between pairs of nitrogenous bases are consistent with those that are generally accepted [62]. Application of the model to the problem of studying the trinucleotide repeat zone allowed us to establish that the frequency of occurrence of open state zones in the CAG repeat tract of the *ATXN2* gene correlates with their number and the age of onset of the disease [26].

The isotopic effects of low concentrations of deuterium in drinking water have long been known [78]. They are well described in a number of review articles, including ours [79,80,81,82]. In article [83], it is described in detail how even single deuterium-to-protium substitutions at key sites affect DNA stability. Furthermore, it is known that increasing deuterium concentration in drinking water even five times above natural levels does not cause toxic effects in the body [84,85,86,87,88]. It should be noted that a limitation of this study is the lack of data on how the deuterium atom enters the CAG pathway. This is the topic of a separate study—how to perform protium–deuterium isotopic exchange at the desired location. This work is devoted to investigating a specific fact: single H/D substitutions in the CAG repeat tract of the *ATXN2* gene increase its stability by reducing the formation of open states, regardless of the position of the deuterium.

## 4. Conclusions

Selective sensitivity to single H/D substitutions in different segments can be observed in DNA molecules under the same external influence [59]. In this study, we observe stabilization of the CAG repeat tract segment in a certain range of torsion effects when a deuterium atom enters the hydrogen bonds between pairs of nitrogenous bases. Single substitutions of protium with deuterium can cause acceleration or deceleration of the reading of genetic information: for example, by changing the rate of hydrogen bonds breaking between pairs of nitrogenous bases. Previously, it was found that single H/D substitutions in the *IFNA17* gene, even outside the coding region, can affect the rate of transcription by changing the probability of OSs occurrence in other parts of the gene [89].

In this work, it was found that regardless of which base pair the deuterium atom falls into in the CAG repeat tract, the probability of additional large OS zones decreases. Accordingly, the stability of the CAG repeat tract increases and the probability of hairpin formation decreases. Hairpins may prevent the reading of genetic information from the glutamine encoding site. It is known that CAA interruptions have a similar but weaker stabilizing effect [37,90].

Note that in the zone of incomplete penetration of the disease, the probability of additional large OSs zone formation decreases by about two times, which may additionally affect a decrease in the rate of elongation of the CAG repeat tract in SCA2. The possibility of such an extension has been noted in a number of works [91,92,93,94].

Many medicinal substances in which hydrogen atoms are replaced by deuterium atoms are promising candidates as drugs, and their new properties are being actively studied [95,96]. So, in 2017, deutetrabenazine was approved for the treatment of Huntington’s disease [97].

Thus, our work creates the preconditions for conducting experimental studies on animals to identify the biological effects of the insertion of a deuterium atom into the hydrogen bonds between nitrogenous base pairs of the CAG repeat tract.

## Figures and Tables

**Table 1:** Coefficients of Equations (1)–(6).

**Type of Base**	**A**	**T**	**G**	**C**
$I\cdot 10^{-44},\;kg\cdot m^{2}$	7.61	4.86	8.22	4.11
R, Å	5.80	4.80	5.70	4.70
$K\cdot 10^{-18}$, N·m	2.35	1.61	2.27	1.54
$k_{12}^{H}\cdot 10^{-2},\;N/m$	6.20	6.20	9.60	9.60
$\beta \cdot 10^{-34},N\cdot m\cdot s$	4.25	2.91	4.10	2.79

## Data Availability

The full dataset is available in the Supplementary Materials.

## Supplementary Materials

The following supporting information can be downloaded at: https://www.mdpi.com/article/10.3390/biomedicines13112708/s1, Figure S1. Calculation results and dynamics of the OS zones formation caused by CAG repeats in the DNA molecule.

## Abbreviations

The following abbreviations are used in this manuscript:

polyQ	polyglutamine tract
H/D	protium/deuterium
OSs	open states
